# Ventilation strategies with different inhaled Oxygen conceNTration during CardioPulmonary Bypass in cardiac surgery (VONTCPB): study protocol for a randomized controlled trial

**DOI:** 10.1186/s13063-019-3335-2

**Published:** 2019-05-03

**Authors:** Meng-Qiu Zhang, Yu-Qi Liao, Hong Yu, Xue-Fei Li, Liang Feng, Xiao-Yun Yang, Hai Yu

**Affiliations:** 1 Department of Anesthesiology, West China Hospital, Sichuan University, Chengdu, 610041 China; 2 Department of Obstetrics and Gynecology, West China Second University Hospital, Sichuan University, Chengdu, China

**Keywords:** Cardiopulmonary bypass, Low tidal volume, Mechanical ventilation, Oxygen concentration, Postoperative pulmonary complications

## Abstract

**Background:**

There is no consensus on the ventilation management during cardiopulmonary bypass (CPB), and the anesthesiologists or the surgeons usually ventilate the lungs with different ventilation strategies or keep them static. Better outcomes are more likely to occur when the ventilation is administered during CPB according to the existing literatures. However, the use of high fraction of inspired oxygen (FiO_2_) is debatable in cardiac surgery. And the potential effects of strategies combining low tidal volume (V_T_) ventilation with different FiO_2_ during CPB on postoperative pulmonary complications (PPCs) are unclear.

**Design:**

The VONTCPB trial is a single-center, prospective, double-blinded, randomized, controlled trial. We are going to recruit total 420 elective cardiac surgery patients with median sternotomy under CPB, who will be equally randomized into three different ventilation strategy groups: NoV, LOV and HOV. (1) The NoV group receives no mechanical ventilation during CPB; (2) the LOV group receives a low V_T_ of 3-4 ml/kg of ideal body weight (IBW) with the respiratory rate (RR) of 10–12 acts/min, and the positive end-expiratory pressure (PEEP) of 5–8 cmH_2_O during CPB; the FiO_2_ is 30%; (3) the HOV group receives a low V_T_ of 3-4 ml/kg of IBW with the RR of 10–12 acts/min, and the PEEP of 5–8 cmH_2_O during CPB; the FiO_2_ is 80%. The primary endpoints are the incidence of the composite of PPCs and the PPCs score. The secondary endpoints refer to the incidence of the oxygenation index (PaO_2_/FiO_2_ ratio) < 300 mmHg at three time points (the moment arriving in the ICU, 6 and 12 h after arrival in the ICU), the surgical incision healing grade, the intubation time, the stay of ICU, the length of hospital stay, and mortality at 30 days after the surgery.

**Discussion:**

The VONTCPB trial is the first study to assess the effects of strategies combining low tidal volume (V_T_) ventilation with different FiO_2_ during CPB on patients’ outcomes.

**Trial registration:**

ChiCTR1800015261. Registered on 20 March 2018.

**Electronic supplementary material:**

The online version of this article (10.1186/s13063-019-3335-2) contains supplementary material, which is available to authorized users.

## Background

Surgical and anesthesia techniques of heart surgery have been greatly improving. However, the postoperative pulmonary complications (PPCs) are still the main complications after surgery, resulting in increasing mortality in cardiac surgery with the incidence of 25% [1]. The PPCs have the forms of respiratory failure, respiratory infection, pleural effusion resulting in thoracentesis, atelectasis, pneumothorax, bronchospasm, aspiration pneumonitis [2]. These complications extend the length of mechanical ventilation and intensive care unit (ICU) stay, indicating worse prognosis [3, 4].

The etiologies of the PPCs are quite complicated. Lungs are isolated from the artificial extracorporeal circulation, which results in blood supply to the lungs only from the bronchial arteries. While two sets of independent circulation systems composing the blood delivery system of the lungs, the bronchial arteries supply only about 3–5% of the pulmonary blood flow system, which may be reduced to one-tenth of the original upon experimental conditions of CPB in pigs model. Those reduced blood flow may be difficult to meet the physiological metabolic needs of the lungs [5]. The tissue of the lungs is also very loose, vascular fluid can be easily transferred to the interstitial tissue under pathologic conditions. Those factors, in addition to the strikes of pulmonary ischemia-reperfusion and reoxygenation, oxygen-free radicals, inflammatory response make the lungs especially vulnerable in the dedicated phases [4].

Perioperative hyperoxia has been declared to bring some benefits. As is known to us all, the increase in the arterial blood partial pressure of oxygen (PaO_2_) could compensate the adverse effects of a reduction in cardiac output on tissue oxygenation. In addition, 80% fraction of inspired oxygen (FiO_2_), hyperoxia can increase the activity of antioxidative enzymes and reduce the oxidative stress induced by ischemia and reperfusion acting similarly to the effect of ischemic preconditioning, thus exerts an active therapeutic effect on organ ischemia-reperfusion injury [6, 7]. However, studies have suggested that hyperoxia associated lung injury and its possible mechanisms [8]. A review suggested that the best usage of hyperoxia is in the timing [9]. Anyway, the debate is still on going.

The common practice of respiratory management during CPB is to suspend the ventilation [10]. Re-expansion pulmonary edema occurs during a long-period collapsed pulmonary reopening, resulting in the reduced lung compliance and ventilation perfusion ratio imbalance [11]. Continuous ventilation during CPB may somehow reduce the pulmonary edema and inflammation. However, the setting of the ventilation parameters is the work of art. A lot of randomized controlled trials (RCTs) were designed to investigate the influence of different ventilation strategies during CPB on the outcomes in heart surgery patients [12–27]. All these RCTs have their own limits. Though the evidence is weak, it still suggests that maintaining mechanical ventilation during CPB is beneficial to lung protection.

The recent meta-analysis made by our research team shows that the ventilation of the resting lung during CPB is indeed effective in improving the oxygenation (PaO_2_/FiO_2_ ratio) and gas exchange. This approach can also reduce the alveolar-arterial oxygen partial pressure difference and the intrapulmonary shunt. But it is unclear of its effect on the long-term prognosis and outcomes for patients. The authors pointed that different FiO_2_ and other respiratory parameters settings may impact the long-term outcomes [28]. An earlier meta-analysis summarized the publications of different ventilation strategies during CPB. Using continuous positive airway pressure (CPAP) and lung recruitment maneuvers can increase oxygenation immediately after CPB to a certain extent, but there is no evidence of a sustained effect [29]. A just published systematic review and meta-analysis concluded that CPAP during CPB may improve the oxygenation and gas exchange while ventilation did not. The authors mentioned that the FiO_2_ had been ignored for a long period [30]. Heinrichs et al. [31] reviewed the RCTs to investigate the impact that hyperoxia has on postoperative organ dysfunction, length of stay, and mortality during adult cardiac surgery. They concluded that the impact of hyperoxia on these outcomes seems to be minimal because of the high heterogeneity of include studies. Similarly, McGuinness et al. [32] failed to demonstrate any difference in AKI, markers of organ damage, or length of stay by comparing the avoidance of arterial hyperoxemia versus usual care during CPB.

There is no generally accepted ventilation management model due to weak evidence of heterogeneity researches [10, 33, 34]. Therefore, we designed a prospective RCT combining the different regimens of ventilation settings during CPB with low or high FiO_2_ to explore the optimal ventilation mode during CPB.

## Objectives

We designed this RCT to compare the clinical effects of three mechanical ventilation strategies on the total incidence of PPCs during postoperative hospitalization. We chose the incidence of composite PPCs as the primary outcome because it is a hard parameter. While in the past many studies only focused on observing biochemical indicators or indirect parameters. You could never tell whether those indicators in the blood have sustained influences in the long run or not. It could be just a transient change after surgery. We hope we would reach a conclusion of optimal ventilation management during CPB, and finally we could make a difference in the clinical practice.

## Methods/design

**Fig. 1:**
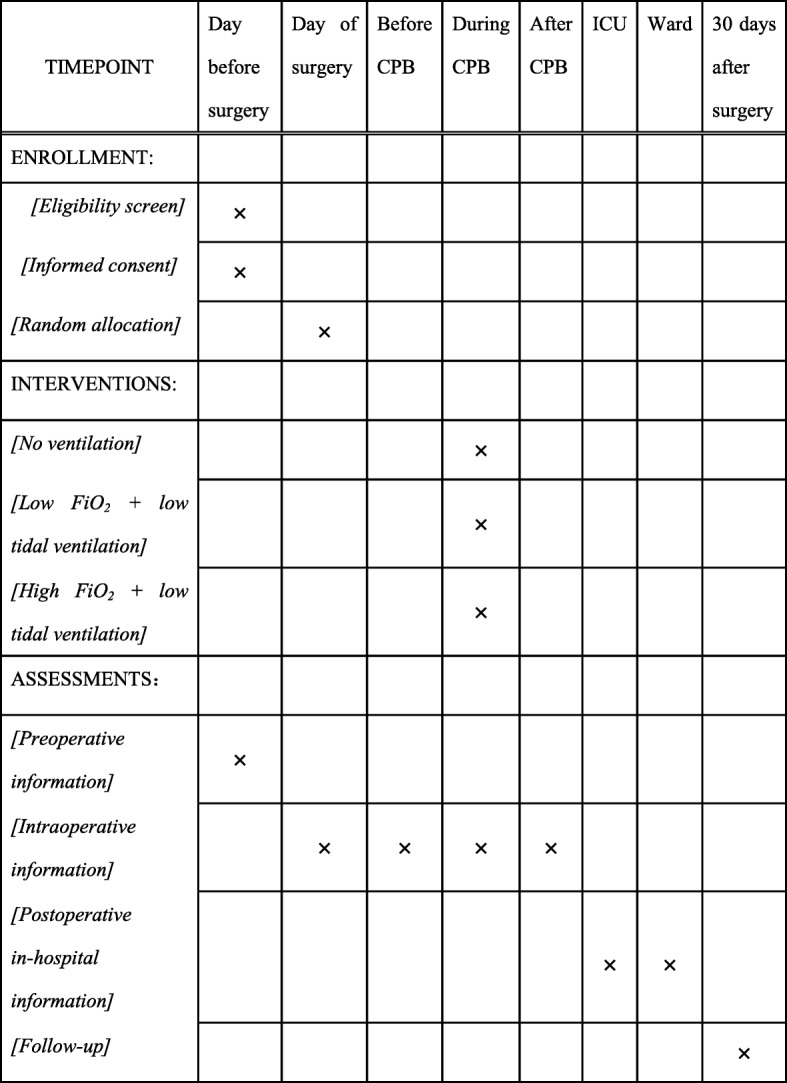
Flow diagram of allocation, intervention, data collection and follow-up

The VONTCPB study is a single-center, double-blinded, three-armed, randomized controlled trial comparing three mechanical ventilation strategies (NoV, LOV and HOV) during CPB in adult patients undergoing cardiac surgery. The protocol structure is written according to the Consolidated Standards of Reporting Trials (CONSORT) 2010 Statement guidelines and follows the Standard Protocol Items: Recommendations for Interventional Trials (SPIRIT) Statement. The SPIRIT checklist can be found in Additional file 1. The SPIRIT figure is illustrated in Fig. 1.

### Ethical approval and trial registration

This clinical study protocol has been approved by the Biomedical Research Ethics Committee of West China Hospital of Sichuan University on 12 March 2018 (Approval number 2017 (400)) and registered in the Chinese Clinical Trials Registry (ChiCTR) (www.chictr.org.cn) with the registration number ChiCTR1800015261 on 19 March 2018.

### Participants

The patients included in the trial must be more than 18 years old and undergo elective cardiac surgery with median sternotomy under CPB. Simultaneous other non-cardiac surgery procedure is excluded. The inclusion and exclusion criteria are shown in Table 1.

**Table 1:** Eligibility criteria

Inclusion criteria	Exclusion criteria
▪ Age ≥ 18 years ▪ Signed informed consent ▪ Elective cardiac surgery patients with median sternotomy under CPB.	▪ Pregnant women ▪ Heart transplantation, ▪ Pulmonary thromboendarterectomy ▪ Aortic arch and other deep hypothermic circulatory arrest surgery ▪ Long-term use of hormone therapy before surgery (≥3 months) ▪ Participated in other similar researches 3 months ago

### Endpoints

The primary endpoints are the incidence of a composite of PPCs and the PPCs score during hospital stay [35–37]. The detailed definitions of PPCs are shown in Table 2. The score is presented in Table 3. Secondary endpoints are listed as follows: the incidence of oxygen index < 300 mmHg at three time points (the moment arriving in the ICU, 6 and 12 h after arrival in the ICU), the surgical incision healing grade, the intubation time, the length of ICU stay and hospital stay, and postoperative 30-day mortality rate.

**Table 2:** Definitions of post-operative pulmonary complications*

Complication	Definition
Respiratory failure	At least one of the following criteria after surgery: ▪ SpO_2_ <90% and requiring oxygen therapy ▪ PaO_2_/FiO_2_ <300 ▪ PaO_2_ <60 mmHg
Respiratory infection	Postoperative antibiotic therapy administered for suspected respiratory infection with at least one of the following criteria: ▪ New or changed sputum ▪ New or changed lung opacity on x-ray ▪ Fever ▪ Leukocyte count >12,000/mL
Pleural effusion	Evidence at chest x-ray of: ▪ Blunting of the costophrenic sinus ▪ In upright position: loss of the sharp silhouette of the ipsilateral hemidiaphragm ▪ In supine position: hazy opacity in 1 hemithorax with preserved vascular shadows ▪ Displacement of adjacent anatomic structures
Atelectasis	Evidence at chest x-ray of: ▪ New parenchymal opacification ▪ Shift of the median structures (mediastinum, hilum or hemidiaphragm) toward the affected area ▪ Compensatory overinflation of contralateral lung
Aspiration pneumonitis	Inhalation of gastric content in the perioperative period with subsequent acute lung injury
Broncho spasm	New expiratory wheezing responsive to treatment with bronchodilators
Pneumothorax	At chest x-ray, presence of air within pleural space, with no vascular bed surrounding the visceral pleura

*Adapted from Bignami et al. [2]

**Table 3:** Postoperative pulmonary complications score

Grade	Definition
Grade 1	- Cough, dry - Microatelectasis: abnormal lung findings and temperature > 37.5 °C without other documented cause; normal chest radiograph - Dyspnea, not due to other documented cause
Grade 2	- Cough, productive, not due to other documented cause - Bronchospasm: new wheezing or pre-existent wheezing resulting in a change in therapy - Hypoxemia (SpO_2_ ≤ 90%) at room air - Atelectasis: gross radiological confirmation (concordance of 2 independent experts) plus either temperature > 37.5 °C or abnormal lung findings - Hypercarbia (PaCO_2_ > 50 mmHg), requiring treatment
Grade 3	- Pleural effusion, resulting in thoracentesis - Pneumonia: radiological evidence (concordance of 2 independent experts) plus clinical symptoms (two of the following: leucocytosis or leucopenia, abnormal temperature, purulent secretions), plus either a pathological organism (by Gram stain or culture), or a required change in antibiotics - Pneumothorax - Noninvasive ventilation, strictly applied to those with all of the following: a) oxygen saturation (SpO_2_) lower than 92% under supplemental oxygen; b) need of supplemental oxygen > 5 L/min; and RR ≥ 30 bpm - Re-intubation postoperative or intubation, period of ventilator dependence (non-invasive or invasive ventilation) ≤ 48 h
Grade 4	- Ventilatory failure: postoperative ventilator dependence exceeding 48 h, or reintubation with subsequent period of ventilator dependence exceeding 48 h
Grade 5	- Death before hospital discharge

### Randomization/Blinding

The random number table was generated by Microsoft Excel in a 1:1:1 treatment ratio. The investigator who is responsible for grouping visits the patients the day before surgery and gets the consents. Once the patient is qualified, he determines the serial number and random number of the patient and writes on the Case Report Form (CRF). He will also inform the anesthesia care providers who do not participate in the study the treatment of the patient. The patient, the data collector and the intensivist are not aware of the treatment information in the whole process. Any intraoperative event (such as pleura incision) or deviation from the protocol is recorded on the CRF.

### Intervention

**Fig. 2:**
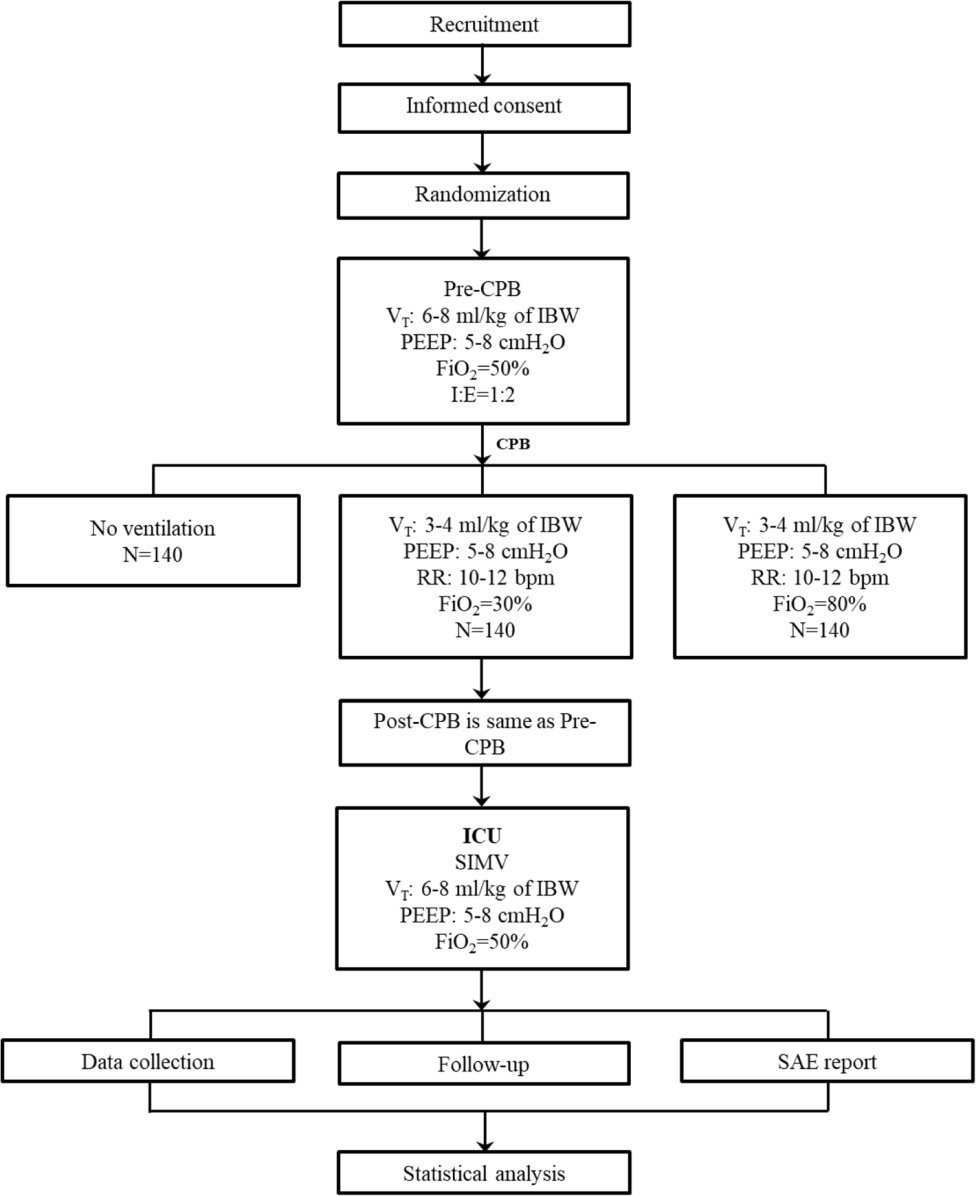
Flow chart. Abbreviations: CPB: cardiopulmonary bypass, V_T_: tidal volume, IBW: ideal body weight, PEEP: positive end-expiratory pressure, RR: respiratory rate, FiO_2_: inspired oxygen fraction, SIMV: synchronized intermittent mandatory ventilation, SAE: severe adverse effect

The participants will be randomly divided into the following three groups (Fig. 2):

1. The NoV arm: no ventilation during CPB.
2. The LOV arm: participants will receive a low V_T_ of 3-4 ml/kg of IBW with the RR of 10–12 acts/min, and the PEEP of 5-8cmH_2_O during CPB; the FiO_2_ is 30% with a flow of 2 L/min.
3. The HOV arm: participants will receive a low V_T_ of 3-4 ml/kg of IBW with the RR of 10–12 acts/min, and the PEEP of 5-8cmH_2_O during CPB; the FiO_2_ is 80% with a flow of 2 L/min.

FiO_2_ 80% will be applied during oxygenation for denitrogenation 5 min before endotracheal intubation. The lung protective ventilation strategy [34, 37–39] will be applied in the participants before and after CPB: V_T_ = 6-8 ml/kg IBW, PEEP = 5–8 cmH_2_O, inspiration/expiration time ratio(I:E) = 1:2, RR = 10–12 to maintain end-tidal carbon dioxide partial pressure (P_ET_CO_2_) between 35 and 45 mmHg, and the FiO_2_ is set to 50%, maintaining pulse oxygen saturation (SpO_2_) ≥95%, with a flow of 2 L/min. If the SpO_2_ cannot be maintained, the anesthesiologists firstly improve patient oxygenation by changing ventilation parameters and ventilation strategies, such as the application of lung alveolar recruitment maneuver (ARM), and then consider increasing the FiO_2_. ARM is performed in all participants at the end of CPB, with manual ventilation creating the airway pressure at 30 cmH_2_O for at least 5 s. Vasoactive drugs will necessarily be medicated if the hemodynamics is affected by the ARM. Maintaining mixed venous oxygen saturation (SvO_2_) ≥ 60% during the CPB (70%–80% would be preferred).

The synchronized intermittent mandatory ventilation (SIMV) mode will be applied once the participants transferred to the ICU. The ventilator parameters settings are as follow: V_T_ = 6-8 ml/kg of IBW, PEEP = 8cmH_2_O, I: E = 1:2, FiO_2_ = 50%. FiO_2_ is adjusted according to the PaO_2_ by performing arterial blood gas (ABG) analyses: if PaO_2_ ≥ 150 mmHg, it is decreased by 10%; but if PaO_2_ ≤ 80 mmHg, then it is increased by 10%; minimum FiO_2_ is 30%. Any additional ARM will be recorded. Other therapeutic approaches and decisions are up to the ICU physicians.

### Perioperative management and monitoring

All these participants will be managed and monitored following the general standard of the practices of the Department of Anesthesiology, West China Hospital of Sichuan University no matter which group they belong to. Intraoperative standard monitoring includes capnography, electrocardiogram (ECG), pulse oximeter, invasive arterial blood pressure, central venous pressure, urine output, nasopharyngeal temperature, transesophageal echocardiogram (TEE), activated clotting time (ACT). Other managements are optional, including bispectral index (BIS) and cerebral oxygen saturation monitoring.

The anesthesiologists give the participants heparin (3 mg/kg) to obtain an ACT of 280 s for aortic cannulation and 480 s before starting the CPB. At the end of the CPB, protamine is given to antagonize heparin with 1:1. The target ACT is about the same as the baseline value. Mean arterial pressure will be kept between 50 and 70 mmHg during CPB.

### Data collection

All the related data are collected on the CRF. Preoperative data include age, height, weight, EuroSCORE II, smoking status, diabetes mellitus, peripheral arterial disease, pulmonary comorbidity, pulmonary infection in the past 30 days and the classification of American Society of Anesthesiologists (ASA). Intraoperative data include type and duration of surgery procedure, duration of CPB and aortic cross clamp, units of allogeneic red blood cell transfusions, volume and type of fluids administered. Postoperative data include time to extubation, need for non-invasive ventilator, re-intubation, postoperative hospital stay, temperature, leukocyte count and chest X-ray. Other data at three time points (the moment arriving in the ICU, 6 and 12 h after arrival in the ICU) include PaO_2_, FiO_2_, and partial pressure of carbon dioxide (PaCO_2_). PPCs events are traced throughout the hospitalization.

### Statistical considerations

#### Sample size

Sample-size calculation is based on two-sided alpha error of 0.05 and 80% power. We performed a pilot-study, showing that the P_max_ is 40% in the LOV group (15 patients) and the P_min_ is 22% in the NoV group (14 patients). Therefore, we expect a reduction of 45% in the incidence of this parameter, and then calculate the sample size of 127 patients per group (381 in total). Considering a dropout fraction of 10%, we will finally need a total of 420 participants.

#### Data analysis

Data will be analyzed following the intention-to-treat principle using SPSS version 22.0 (SPSS, Chicago, IL, USA). Comparisons in the dichotomous variables will be made by the chi-square tests (or Fisher exact tests where numbers are small). Comparisons in the continuous variables will be made by analysis of variance or the nonparametric Kruskal-Wallis H test. Two-sided significance tests will be used throughout. The Results will be presented as n (%), mean (SD), or median (interquartile range). Bonferroni correction will be used when doing post hoc analysis.

## Discussion

About 1–1.25 million patients undergo cardiac surgery worldwide each year and the number continues to increase [40]. CPB is an essential part of major cardiac surgery which may induce PPCs after operation. Since the PPCs are leading causes of mortality and morbidity [37], the effective preventive measures are particularly important and urgently needed. Various researches have been performed to reach this goal. The MECANO trial (NCT03098524) aims to assess the effects of mechanical ventilation (V_T_: 3 ml/kg, PEEP: 5cmH_2_O, RR: 5 bpm) on all-cause mortality and respiratory failure comparing with no ventilation during CPB [41]. It does not provide the detailed usage of oxygen though the ambitious number of patients (*n* = 1500). The CPBVENT trial (NCT02090205) is a three-armed multicenter study [42], which compares two different ventilation strategies (CPAP: 5cmH_2_O or V_T_: 2-3 ml/kg of IBW, PEEP: 3-5cmH_2_O, RR: 5 bpm) with no ventilation during CPB. This trial’s primary endpoint (PaO_2_/FiO_2_ ratio) can be easily identified, observed, and calculated, but indirect. That is why we use it as a secondary endpoint. Besides, the inclusion and exclusion criteria are relatively strict, making the conclusion only applicable to a specific group of populations. The purpose of the VONTCPB study is to determine the impact that the different FiO_2_ has on the PPCs when combining the ventilation strategy with low V_T_(3-4 ml/kg of IBW, only about half of the normal tidal volume), routine respiratory rate (10–12 bpm) and PEEP (5–8 cmH_2_O) during CPB. This trial is the first study to investigate the effect of oxygen under different ventilation strategy during CPB. The number of the sample size (*n* = 420) is adequate. The primary endpoints are clinically relevant. The purpose of setting ventilator parameters in this way is to make the ventilation closer to the general. And this ventilation management during CPB may potentially benefit patients’ outcomes, but at the same time it has an imperceptible impact on the operating field.

There are limitations of the trial due to the inherent properties such as its single-center design. It is questionable whether the conclusion drawn by the VONTCPB trial can be applied in other hospitals because of the different clinical practice in other hospitals. Besides, only the volume controlled ventilation mode is applied, so it is unknown whether it is also applicable in other ventilation mode, such as pressure controlled mode.

In general, the VONTCPB trial would answer the question that whether maintaining mechanical ventilation during CPB is more preferable than no ventilation. Better still, which level of FiO_2_ is beneficial to patients will be determined from this trial.

## Trial status

The trial was started after we obtained the approval of local ethics committee and registered in the Chinese Clinical Trials Registry. We plan to spend six months to include cases and complete the trial in November 2018.

## Additional file

Additional file 1: The SPIRIT 2013 checklist of this trial. (DOCX 53 kb)

## Abbreviations

ABG: Arterial blood gas
ACT: Activated clotting time
ARM: Alveolar recruitment maneuver
ASA: American Society of Anesthesiologists
BIS: Bispectral index
CPAP: Continuous positive airway pressure
CPB: Cardiopulmonary bypass
CRF: Case report form
ECG: Electrocardiogram
FiO_2_: Inhaled oxygen concentration
IBW: Ideal body weight
ICU: Intensive care unit
PaCO_2_: Partial pressure of carbon dioxide
PaO_2_: Arterial blood partial pressure of oxygen
PEEP: Positive end-expiratory pressure
P_ET_CO_2_: End-tidal carbon dioxide partial pressure
PPCs: Postoperative pulmonary complications
RCTs: Randomized controlled trials
RR: Respiratory rate
SIMV: Synchronized intermittent mandatory ventilation
SpO_2_: Pulse oxygen saturation
SvO_2_: Mixed venous oxygen saturation
TEE: Transesophageal echocardiogram
V_T_: Tidal volume
